# Lifestyle, cardiovascular risk knowledge and patient counselling among selected sub-Saharan African family physicians and trainees

**DOI:** 10.4102/phcfm.v11i1.1701

**Published:** 2019-03-26

**Authors:** Pius O. Ameh, Kenneth Yakubu, Miriam Miima, Olugbemi Popoola, Gulnaz Mohamoud, Klaus B. von Pressentin

**Affiliations:** 1 Department of Accident and Emergency, Federal Medical Centre, Keffi, Nigeria; 2 Department of Family Medicine, University of Jos, Jos, Nigeria; 3 Columbia Africa Healthcare Limited, Nairobi, Kenya; 4 Department of Family Medicine, Federal Medical Centre, Abuja, Nigeria; 5 Department of Family Medicine, The Aga Khan University Hospital, Nairobi, Kenya; 6 Division of Family Medicine and Primary Care, Stellenbosch University, Stellenbosch, South Africa

## Abstract

**Background:**

Cardiovascular disease (CVD)-related deaths in sub-Saharan Africa (SSA) are on the rise, and primary care physicians could facilitate the reversal of this trend through treatment and prevention strategies.

**Aim:**

The aim of this study was to determine the relationship between physician lifestyle practices, CVD prevention knowledge and patient CVD counselling practices among family physicians (FPs) and family medicine (FM) trainees affiliated to FM colleges and organisations in SSA.

**Setting:**

FPs and FM trainees affiliated to FM colleges and organisations in Anglophone SSA.

**Methods:**

A web-based cross-sectional analytical study was conducted using validated, self-administered questionnaires. Following collation of responses, the relationship between the participants’ CVD prevention knowledge, lifestyle practices and CVD counselling rates was assessed.

**Results:**

Of the 174 participants (53% response rate), 83% were married, 51% were females and the mean age was 39.2 (standard deviation [SD] 7.6) years. Most of the participants responded accurately to the CVD prevention knowledge items, but few had accurate responses on prioritising care by 10-year risk. Most participants had less than optimal lifestyle practices except for smoking, vegetable or fruit ingestion and sleep habits. Most participants (65%) usually counselled patients on nutrition, but less frequently on weight management, exercise, smoking and alcohol. The region of practice and physicians with poor lifestyle were predictive of patient counselling rates.

**Conclusion:**

Training on patient counselling and self-awareness for CVD prevention may influence patient counselling practice. Promoting quality training on patient counselling among FPs as well as a healthy self-awareness for CVD prevention is thus needed. The complex relationship between physician lifestyle and patient counselling warrants further study.

**Keywords:**

family physicians; cardiovascular diseases; lifestyle counselling; sub-Saharan Africa; online survey; family medicine trainee.

## Introduction

Non-communicable diseases (NCDs) are on the rise globally.^1^ In 2012, stroke and coronary heart disease accounted for more than 31% of deaths from all causes, making them the leading cause of death worldwide.^1^ In low- and middle-income countries (LMICs), NCDs accounted for 13 million deaths of people under 70 years of age in 2013.^1^ Sub-Saharan Africa (SSA) has not been spared in this pandemic. Approximately 1 million deaths in SSA in the year 2013 were attributed to cardiovascular diseases (CVDs), representing an 81% increase from 1990 levels.^2^ This increase was observed for all component CVDs, ranging from a 7% increase because of rheumatic heart disease to 185% and 196% increases because of peripheral arterial disease and atrial fibrillation, respectively.^2^ As of 2014, the prevalence of hypertension and diabetes in SSA had reached 10–20 and 14.7 million, respectively.^3^

The prevention and management of the increasing burden of NCDs must be regarded as a global priority. Family physicians (FPs) are essential in carrying out the individual or group prevention and treatment strategies necessary for CVD risk reduction, as they are frequently the first point of contact in health systems.^4^ They are concerned with continuing responsibility for total health care of individuals and families: from first contact acute care to care for chronic conditions as well as preventive and promotive care.^4^ These responsibilities should motivate FPs to deliver quality health care by applying the best evidence to patients’ problems.^4^

In line with the above, the World Health Organization (WHO) and the International Society for Hypertension (ISH) recommend that primary care workers use WHO or ISH risk prediction charts to screen and counsel for CVD risk factor reduction in low-resource regions such as SSA.^5^ These charts indicate 10-year risk of a fatal or nonfatal major cardiovascular event (e.g. myocardial infarction or stroke), according to age, sex, blood pressure, smoking status, total blood cholesterol and the presence or absence of diabetes mellitus for 14 WHO epidemiological subregions.^5,6^ The 14 epidemiological subregions were created based on mortality strata of the low-, middle- and high-income countries of the world, and each region has a specific risk prediction chart that should be applied in that region alone.^5^ For instance, the charts developed for SSA were designed to be applied without the need for cholesterol, unlike in high-income settings. The charts provide approximate estimates of CVD risk in people who do not have established coronary heart disease, stroke or other atherosclerotic disease. Once those at high cardiovascular risk have been identified, the charts are then used to motivate dietary modifications, daily physical activities, smoking cessation and weight control. When appropriate, they could also be used to guide initiation or continuation of aspirin, antihypertensive and lipid-lowering drugs.^5^

Currently, the best evidence available appears to support counselling for CVD prevention. A systematic review by the United States Preventive Services Task Force (USPSTF) in 2014 found that in patients with established CVD risk factors, combined intensive lifestyle counselling (diet and/or physical activity) reduced total cholesterol, low-density lipoprotein, fasting blood glucose, blood pressures, diabetes incidence and weight.^7^ These findings were most robust at 12–24 months after the interventions.^7^ Combined intensive lifestyle counselling also improved behavioural outcomes, that is, objectively measured and self-reported dietary changes and physical activity events at 12–24 months.^7^ However, only one randomised controlled trial showed reduced CVD events and mortality at 6.6 years following intensive combined lifestyle counselling with a medication protocol which included seven counselling sessions over a 4-month period. Other studies measuring CVD events and mortality at 10 years of follow-up did not find any benefit, although the CVD event rates were generally low in the study populations of the reviewed articles. The USPSTF concluded that the applicability of these findings depended on the availability of intensive counselling practice and patient adherence.^7^ The USPSTF also recommended, based on the challenges they faced in performing the systematic review, that further randomised controlled studies on behavioural interventions should follow a standardised approach to population selection, intensity of intervention and outcome measures so that subsequent syntheses of evidence could be more robust.^8^

Primary care physicians (PCPs) play an important role in patient counselling for CVD prevention and can support patients as they initiate and adhere to lifestyle changes. However, some barriers to CVD risk assessment and counselling behaviour have been identified, as even in developed regions physicians frequently do not effectively implement counselling and CVD risk reduction behaviour.^9^ Studies among PCPs in Europe indicate a wide variation in this practice with between 20% and 86% of clinicians providing patient counselling on CVD prevention.^10,11^ In the United States, physician specialty was shown to affect awareness and use of recommended guidelines in CVD prevention. In this study, PCPs were more likely to incorporate CVD prevention guidelines than other specialists in routine care.^9^ In addition, a physician’s lifestyle counselling (for CVD) has been linked to his personal lifestyle habits – even with those habits established during his undergraduate training years – at least in the context of physical exercise.^12^ Other factors known to affect CVD risk assessment and counselling behaviour among physicians include inadequate compensation or reward and inadequate resources and/or time.^13^

Similar to the developed regions, some studies have been conducted among PCPs in SSA. A study in South Africa revealed that different lifestyle modification knowledge exists among physicians and students practising in primary healthcare centres and tertiary centres.^14^ Another study revealed that trainers lacked confidence in the effectiveness of behaviour change counselling and in approaches to training.^15^ Yet, little is known about the influence of personal health-related lifestyle practices and knowledge of CVD prevention on patient counselling behaviour among FPs or family medicine (FM) trainees in SSA. This study is aimed at filling this gap in the literature by determining the relationship between physician health-related lifestyle practices, physician knowledge of CVD prevention and patient counselling practice among FPs and FM trainees in SSA. Specifically, the objectives were to (1) assess the knowledge of CVD prevention among FPs and FM trainees, (2) assess their personal health-related lifestyle practices, (3) assess lifestyle counselling and CVD screening frequency among FPs and FM trainees in SSA and (4) determine the relationships between the former two and the latter concepts.

## Research methods and design

### Study design

This was an online, cross-sectional analytical study.

### Study setting

This study focused on the SSA region, defined as all of Africa excluding North Africa.^16^ The FP and FM trainees’ hospital catchment area was diverse, reflecting an urban–rural population mix.

### Study population

This study comprised all FPs (i.e. doctors who had completed a residency training in FM) and FM trainees who were practising or training in Anglophone SSA. Their contact details were drawn from the Faculty of Family Medicine, West African College of Physicians (WACP) and the Society of Family Physicians of Nigeria (SOFPON), the online platforms of the South African Academy of Family Physicians, South African Family Medicine Registrars and the African Primary Care and Family Medicine Education Network (PRIMAFAMED), World Organisation of National Colleges, Academies and Academic Associations of Family Physicians/General Practitioners (WONCA) African Region and AfriWon Renaissance (the WONCA Young Doctor Movement for Africa). Survey links were also sent through volunteers to the group email addresses of FPs and FM trainees in East Africa.

### Inclusion criteria

Family physicians and FM trainees practising in SSA at the time of the study with Internet access were included in the study.

### Exclusion criteria

Family physicians and FM trainees who withdrew consent, had inconsistent online access or were not contacted for various reasons, for example, annual leave, were excluded from the study.

### Sample calculation

Based on the highest proportion obtained from a similar survey among general practitioners (GPs) in Germany,^17^ the anticipated frequency of FPs and FM trainees, who always counsel patients on prevention of CVD, was set at 69.7%. The following details were entered into the Open Epi section on sample size for frequency in an unknown population: population size (*N*, with finite population correction factor) of 1 000 000; hypothesised percentage frequency of the outcome factor in the population (*p*): 69.7% ± 5; confidence limits (*d*): 0 (absolute ± %): 5%; and confidence level (%): 95. This yielded a minimum sample size of 325.

### Sampling technique and recruitment

A stratified random sampling technique was used for this study. The existing strata were Central, East, West and Southern Africa. For all participants that opted in to the study and met the inclusion criteria, an equal proportion of 82 trainees and specialists from each of the existing strata were to be recruited via simple random sampling technique to give a total sample of 328.

### Study tools

A self-designed web-based questionnaire was used and consisted of an introductory section, a consent form and the main questionnaire.^18^ The introductory section provided a brief background to the study and its aim. The main questionnaire elicited baseline characteristics of the participants, assessed physicians’ knowledge of CVD prevention based on the 2007 WHO guidelines, physicians’ personal health-related lifestyle practices and their CVD counselling and screening practices.^5^ (See Appendices 4 and 5)

### Reliability and validity of the study tools

The section assessing physician knowledge of CVD prevention was also self-designed by the authors based on the 2007 WHO guidelines on prevention of CVD.^5^ Sixteen questions were derived from the 12 recommendations of the guideline using either a Likert scale or multiple-choice format. The focus was to test the three basic cognitive levels of learning as prescribed in Bloom’s taxonomy of learning (i.e. knowledge, comprehension and application).^19^ After the initial item generation for the questionnaire, 12 specialists considered to be experts in the management of CVD (i.e. five FPs, an internist, a lifestyle specialist, an endocrinologist, a public health specialist, a health educator and two cardiologists), from the affiliate institutions of the authors, assessed each question for relevance and clarity. Their input was used to modify the questionnaire. The final item content validity index (I-CVI) was 1 for the 16 questions. Average scale content validity index (S-CVI/Ave) was 0.98 for relevance and 0.93 for clarity. Calculation of I-CVI and S-CVI was based on the formula by Zamanzadeh et al.^20^ For this tool, the maximum physician knowledge of CVD prevention score was 49.

The sections on physician personal health-related lifestyle practice and CVD counselling practice were adapted from a similar study by Voltmer et al.^17^ The items were derived from questionnaires used in a German national health survey and other international surveys of physicians.^17^ A total of 14 items were adapted for the physician personal health-related lifestyle practices and eight items for the CVD counselling practice.

The section assessing baseline characteristics (including age of participants, sex, designation, etc.) was self-designed with face validity assured, following a literature review of unique characteristics of participants that may have a direct effect on CVD counselling practice. It consisted of 21 items consisting of multiple-choice and open-ended questions that required short answers. Combining this section with the sections on personal health-related lifestyle practice and CVD counselling practice would yield 43 items. The combined sections were also reviewed by nine different physicians (four FPs and five non-FM specialists) who worked in the same institutions with the authors and were also considered to be experts in the management of CVDs. The 43 items (reflecting baseline characteristics of participants, their personal health-related lifestyle practices and patient counselling practices) were retained and these had an I-CVI ranging from 0.7 to 1.0. S-CVI/Ave was 0.95 for relevance and 0.90 for clarity. Calculations were also based on the formula by Zamanzadeh et al.^20^ The maximum scores for physician personal health-related lifestyle practice and CVD screening or patient counselling practices were 49 and 35, respectively.

### Data collection

The survey was administered using the SurveyMonkey^©^ platform and survey links sent to the email groups of the Faculty of Family Medicine, WACP and the SOFPON, the online platforms of the PRIMAFAMED, WONCA African Region and AfriWon Renaissance. Survey links were also sent through volunteers to the group email addresses of FPs and FM trainees in East Africa. Once the study period elapsed, the link was closed and all data collated.

### Data analysis

Primary outcome variables included: (1) frequency of participants with adequate CVD counselling practice scores, defined for the purposes of this study, as always or usually counselled items combined and (2) the CVD and counselling practice scores. Secondary outcome variables included CVD prevention knowledge scores, proportion of participants who got each question on CVD prevention correct, proportion of participants with optimal personal health-related lifestyle practices, physician personal health-related lifestyle scores, frequency distribution of participants by sex, marital status, job designation, the presence of CVD risk among participants and among their friends or family members, type of community served by respondent’s practice, region of practice, mean age, median time at work and median monthly income.

The Pearson’s test of correlation was used to test for association between CVD screening or counselling practice scores and secondary variables that were continuous, while χ^2^ test of independence was used to test for association between the proportion of participants with optimal CVD counselling practice and secondary outcome variables that were categorical. Logistic regression was then used to test for the relationship between the primary and secondary outcome variables that showed statistically significant association. Data collation, description and analyses were done using SPSS version 23 and Microsoft Excel software^21,22^; *p* was set at 0.05.

### Ethical considerations

Ethical clearance was granted by the Health Research Ethics Committee, Federal Medical Centre, Keffi, Nasarawa State (Ethical Clearance Reference Number FMC/KF/HREC/078/15, Registration Number /21/12/2012). Informed consent was sought from each participant on the SurveyMonkey^©^ platform by having them complete the consent form (see Appendix 5). Password protection and other security methods were used to protect confidential information.

## Results

The total number of participants was 174 (yielding a response rate of 53%), most of whom were married (145/174, 83%), females (89/174, 51%) with a mean age of 39.2 years. The majority of the participants (64/174, 37%) were FM trainees who had been in the residency programme for more than 2 years, mostly practising in West Africa (103/174, 59%) and were urban based (71/174, 41%) (see Table 1).

**TABLE 1:** Demographic characteristics of participants.

Characteristic	Frequency†	Percentage
Sex (*N* = 174)		
Male	75	43.1
Female	89	51.1
Marital status (*N* = 164)		
Married	145	83.3
Single	18	10.3
Separated	1	0.6
Region of practice or training (*N* = 174)		
West Africa	103	59.2
Southern Africa	33	19.0
East Africa	28	16.1
Central Africa	1	0.6
Community type served by practice (*N* = 174)		
Urban	71	40.8
Both urban and rural	43	24.7
Semi-urban	26	14.9
Rural	24	13.8
Designation (*N* = 174)		
FM resident (> 2 years in training)	64	36.8
FM specialist (≤ 5 years since completing training)	46	26.4
FM specialist (> 5 years since completing training)	33	19.0
FM resident (≤ 2 years in training)	21	12.1

FM, family medicine.

† , Missing values not included in the frequency.

The most common self-reported CVD risk factor among the participants was hypertension, affecting 9.2% (16/174) of them, while 2.9% (5/174) had experienced either angina or a myocardial infarction. Most of the participants (106/174, 61%) had at least one family member with a cardiovascular risk factor and 35.0% (61/174) had family members with a history of a CVD (see Table 2).

**TABLE 2:** Characteristics of participant cardiovascular disease risk factors.

Characteristic	Frequency	Percentage
Respondent cardiovascular risk		
Hypertension	16	9.2
Obesity	13	7.5
Dyslipidaemia	6	3.4
Diabetes	5	2.9
Respondent cardiovascular disease		
Angina/MI	5	2.9
Stroke	1	0.6
PE/DVT	1	0.6
PAD	1	0.6
Relative with at least one CVD risk factor	106	61
Relative with a history of cardiovascular disease	61	35.1

CVD, cardiovascular disease; PE/DVT, Pulmonary Embolism/Deep Vein Thrombosis; PAD, Peripheral Arterial Disease; MI, Myocardial Infarction.

The median scores for respondent’s knowledge of CVD prevention, personal health-related lifestyle and CVD counselling practice were 10, 30 and 14.6 respectively. Comparing the 25 and 75 percentiles with the maximum scores obtainable for each of these outcomes, most of the participants showed appropriate CVD prevention knowledge and personal health-related lifestyle scores with less than optimal patient CVD counselling practice (see Table 3).

**TABLE 3:** Distribution of participants’ scores for personal health-related lifestyle, knowledge of cardiovascular disease prevention and patient counselling practice.

Questionnaire category	Maximum score obtainable	25th Percentile	75th Percentile	Median
Physician personal health-related lifestyle score	49.0	27.0	32.0	30.0
Knowledge of CVD prevention score	16.0	10.0	10.0	10.0
Patient counselling practice score	35.0	12.5	17.0	14.6

CVD, cardiovascular disease.

**FIGURE 1:**
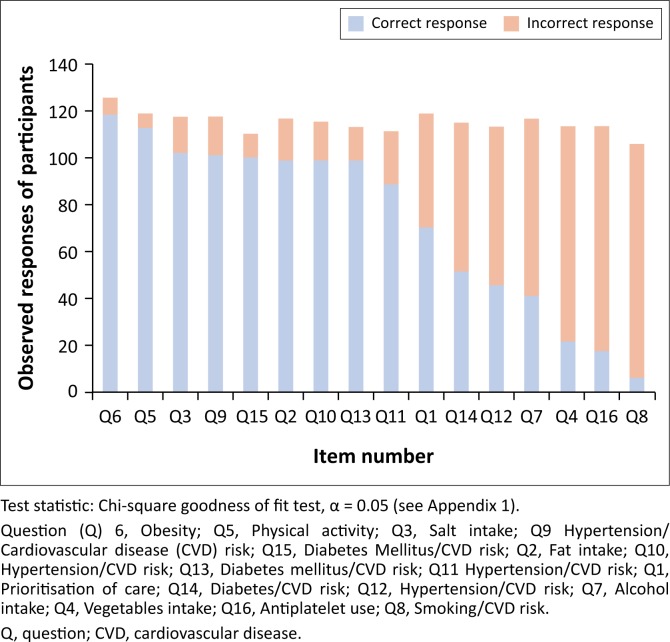
Knowledge of cardiovascular event prevention: Distribution of accuracy of responses from most accurate to least accurate.

Concerning knowledge of CVD prevention, the majority of the participants (118/174, 68%) had accurate responses for question 6 (management of obesity), a relatively similar proportion of participants (57% – 65%) had accurate responses for questions on physical activity (Q5), salt intake (Q3), hypertension or CVD risk (Q9, Q10), diabetes mellitus or CVD risk (Q15, Q13) and fat intake (Q2). A marked decline for accurate responses was observed for other questions with the least being proportions of participants with accurate responses for question 8 (smoking and CVD risk). This distribution was statistically significant (see Figure 1).

**FIGURE 2:**
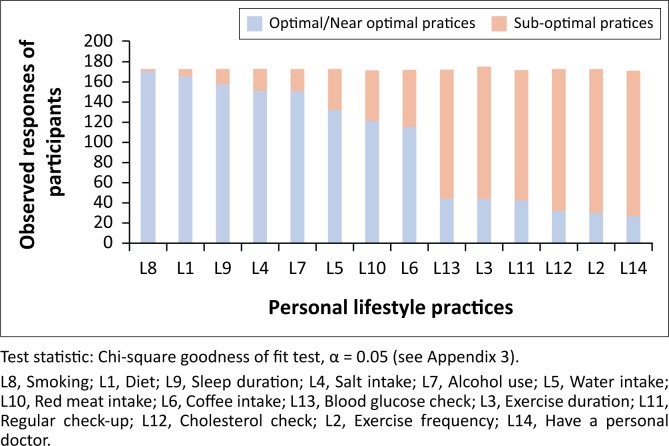
Distribution of personal lifestyle practices of study participants, from most optimal to least optimal.

For the distribution of personal health-related lifestyle practices, almost all of the participants (173/174, 99%) had optimal or near-optimal lifestyle practice for item L8 (smoking). Similar proportions, 96% and 92% were seen for items L1 (diet) and L9 (sleep duration), respectively. A progressive decline of proportions of participants with optimal lifestyle practices was seen for majority of the other items with the least being item L14 (having a personal doctor), 16% (28/174). This distribution was statistically significant (see Figure 2).

**FIGURE 3:**
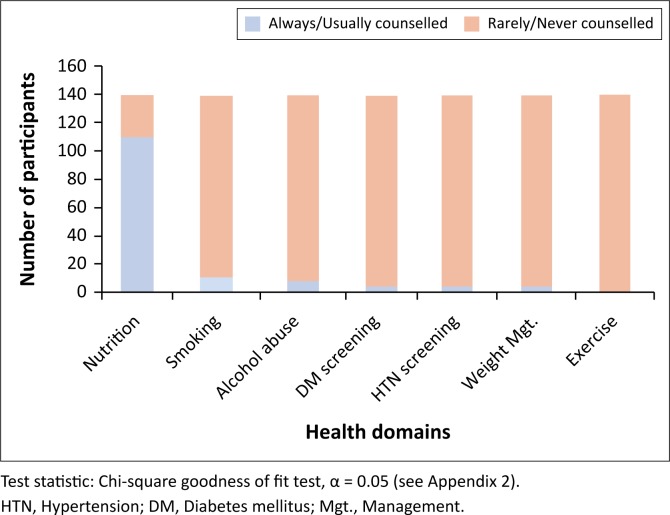
Distribution of participants by frequency of counselling practice in various health domains.

Concerning frequency of patient CVD counselling practice, most of the participants (113/174, 65%) always or usually counselled their patients on nutrition, while the proportions of those who always or usually counselled their patients on smoking, alcohol abuse, weight management, diabetes and hypertension screening ranged from 2% to 6% with the least being counselling for exercise, 0.6% (1/174). This distribution was also statistically significant (see Figure 3).

A negative correlation was observed between patient CVD counselling practice and physician personal lifestyle scores, *R* = -0.3 and *p* < 0.01 (Table 3). When classified by region, Southern Africa had the highest proportion of participants (15/30, 50%) with adequate frequency of CVD counselling practice, while those from West Africa had the lowest, 9% (8/87) (see Table 4).

**TABLE 4:** Pearson’s correlation between total counselling score and total lifestyle score.

Imputation number	Total CVD counselling practice score		Total physician personal lifestyle score	
	Pearson correlation	Sig. (two-tailed)	Pearson correlation	Sig. (two-tailed)
**Pooled**				
Total CVD counselling practice score	1.00	-	−0.31*	< 0.01
Total physician personal lifestyle score	−0.31*	< 0.01	1.00	-

CVD, cardiovascular disease; Sig., significance.

* , Significance level α = 0.05.

Also, most of the participants (11/19, 58%) who practised or trained in a rural setting had an adequate frequency of CVD counselling practice, while those in urban settings had the least proportion (9/60, 15%) of participants with an adequate frequency of CVD counselling practice (see Table 5).

**TABLE 5:** Association between cardiovascular disease counselling practice and type of community served by the participants

Community type served	CVD counselling practice				Total	*p*-value*
	Adequate		Inadequate			
	*N*	%	*N*	%
Urban	9	15.0	51	85.0	60	0.001
Rural	11	57.9	8	42.1	19	
Semiurban	4	16.7	20	83.1	24	
Both urban and rural	7	18.9	30	81.1	37	

**Total**	**109**	**-**	**31**	**-**	**140**	**-**

Original data: Listwise, *N* = 140; pooled imputation data: Listwise, *N* = 164.

Adequate, always or usually; inadequate, rarely or never.

CVD, cardiovascular disease.

* , *χ*^2^ = 16.5, *df* = 3, *α* = 0.01 (two-tailed).

These distributions were statistically significant. Other baseline characteristics of the participants did not show statistically significant associations with CVD counselling practice. There was no difference in the distribution of counselling practice scores between the trainees and the specialists as most of the trainees (66/84, 78.6%) and specialists (70/90, 77.8%) had poor counselling scores (χ^2^ = 0.02, df = 1, *p* = 0.90). From the aforementioned factors with significant associations, only the region of participants’ practice or training and physician personal health-related lifestyle score was predictive of CVD counselling practice score with the latter having the highest OR of 2.96. This was statistically significant (see Tables 6 and 7).

**TABLE 6:** Association between sub-Saharan Africa region and cardiovascular disease counselling practice scores.

SSA region	CVD counselling practice scores				Total	*p*-value*
	Adequate		Inadequate			
	*N*	%	*N*	%
West Africa	8	9.2	79	90.8	87	< 0.01
East Africa	7	33.3	14	66.7	21	
Southern Africa	15	50.0	15	50.0	30	
Central Africa	1	-	1	-	2	

**Total**	**31**	**-**	**109**	**-**	**140**	**-**

Original data: Listwise, *N* = 140; pooled imputation data: Listwise, *N* = 164.

Adequate, always or usually; inadequate, rarely or never.

CVD, cardiovascular disease; SSA, sub-Saharan Africa.

* , *χ*^2^ = 24.4, *df* = 3, *α* = 0.01 (two-tailed).

**TABLE 7:** Logistic regression between grouped cardiovascular disease counselling practice, physician lifestyle, region and type of community type served by the participants.

Predictors	*B*	SE	Wald	df	Sig.*	Exp. (*B*)	95% CI for exp. (*B*)	
							Lower	Upper
Region	1.08	0.27	16.77	1	< 0.01	2.96	1.76	4.97
Community type served	−0.04	0.19	0.03	1	0.85	−0.97	0.66	1.41
Total personal lifestyle score	−0.19	0.05	12.7	1	< 0.01	−0.83	0.74	0.92
**Constant**	**2.26**	**1.58**	**2.04**	**1**	**0.15**	**9.53**	**-**	**-**

Original data: Listwise, *N* = 140; pooled imputation data: Listwise, *N* = 164.

*B*, beta; *SE*, standard error; Sig., significance; *df*, degree of freedom; Exp., Exponent of coefficient; CI, confidence interval.

* , Significance level *α* = 0.01 (two-tailed).

## Discussion

In assessing knowledge of CVD prevention among FPs and FM trainees in SSA, we found that overall most of the participants had accurate responses for the CVD prevention knowledge items. This is similar to findings from the National Study of Physician Awareness and Adherence to Cardiovascular Disease Prevention Guidelines in the United States, which showed a significant level of PCP awareness (90%) on CVD prevention guidelines.^9^ However, despite being relatively well aware of regional guidelines on CVD prevention, our participants were only partially aware of recommendations on prioritisation of care according to a 10-year CVD risk. Our findings are consistent with a United States survey of FPs carried out to assess hyperlipidaemia management as part of the overall CVD risk reduction strategy. They found that even though awareness of ATP III guidelines was high (85%) and most screened for hyperlipidaemia (89%), management of hyperlipidaemia according to CVD risk (and use of a risk calculator) was quite low (17%) among participants.^23^

On the whole, the assessment of personal health-related lifestyle practices revealed that most of the participants had optimal or near-optimal lifestyle practices for the *use of cigarettes, eating salad* or *raw vegetables and fruits*, and also for *sleeping habits*. A progressive decline in the proportions of participants with optimal lifestyle practices for other lifestyle items was observed with the least proportions of participants indicating that they had regular check-ups, exercised frequently or had a personal doctor. These may be linked to the rather low prevalence of cardiovascular risk factors (9%) among the participants in our study, suggesting that smoking, diet and sleeping habits were the factors most associated with the presence of CVD risk in this population. Conversely, a study among Cameroonian PCPs showed that 12.3% of primary care physicians smoked, 61.5% consumed alcohol in excess, 23.1% were obese, 26.2% had hypertension and 54.6% had a family history of CVD despite participants in both studies having the same mean age.^24^ The higher proportion of PCPs with suboptimal lifestyle practices in their study and similar proportion of participants with family history of CVD risk further lends credence to the link between observed protective lifestyle factors and low prevalence of CVD risk factors among our participants.

Concerning CVD counselling, most of the participants (65%) always or usually counselled their patients on nutrition. The proportions of those who always or usually counselled their patients on smoking, alcohol abuse, weight management, diabetes and hypertension screening ranged from 2% to 6% with the least being counselled for exercise, 0.6%. This may be linked to the low proportion of participants with optimal exercise frequency. An audit on the AUSDRISK (Australian type 2 diabetes risk assessment tool) usage by GPs found that lifestyle counselling only occurred in 1.2% of general practice encounters in 2009.^25^ Apart from the counselling rates for nutrition observed in our study, the findings on counselling for other items were comparable to the Australian study. The difference may be explained by the stronger presence of allied healthcare providers and programmes in their CVD prevention programmes as compared to the practice settings of our participants.^26^

Current evidence shows a positive relationship between physician lifestyle practice and counselling practice rates and is in contrast with our findings, which revealed more frequent counselling practices among those with poorer lifestyle habits.^27,28,29^ The relationship between physician personal health-related lifestyle practices and rates of patient counselling may not be as straightforward as suggested. A study among PCPs and nurses affiliated with the Mayo Clinic, USA, showed no relationship between physician lifestyle practices and patient counselling.^30^ It also showed that physicians did not think their patients would respond negatively if they counselled them on health behaviours they also struggled with. Other studies have suggested that being self-aware of the need for lifestyle change as well as experiencing what it means to struggle with poor health behaviour can make physicians more empathetic to their patients who struggle with the same issues, thus increasing counselling rates.^31,32^ This may suggest that the participants in this study recognised their own struggle with lifestyle modification, and this was associated with higher CVD counselling practice rates as compared to those who had optimal lifestyle practices. However, this complex relationship deserves more research.

Having explored factors associated with CVD counselling practice rates, we observed that only *region of participants’ practice* or *training* and *physician personal lifestyle practice were* predictive of patient CVD counselling practice rates with the latter having the highest OR of 2.96. Southern Africa had the highest proportion of participants (15/30, 50%) with adequate CVD counselling practices, while those from West Africa had the lowest, (8/87, 9%) (Table 4). Within the southern African sub-region, a best practice brief behaviour change counselling (BBCC) training programme was recently developed and implemented among PCPs.^33^ To the best of our knowledge, a similar programme has not yet been implemented in the East and West African subregions. Our findings may thus be a reflection of the effect of quality of training and physician awareness of the need for personal lifestyle change on patient counselling rates.

## Limitations or strengths of the study

As an equal proportion of responses could not be obtained from each sub-Saharan region, the results would have to be interpreted with caution. This lack of equivalent responses could be attributed to a disproportion in the number of FPs and FM trainees across the continent as well as the recruitment communication from the various regional organisations. This study did not account for non-English-speaking FPs and FM trainees and FPs and FM trainees without Internet access, therefore we may have missed out on a significant population of FPs and FM trainees. Also, while we assessed for frequency of counselling practice, we could not assess the quality of the counselling practice. In addition, we did not account for differences between public and private sector physician **or** trainees as their differing work burdens, incentives and oversight could affect counselling practices.

Despite the drawbacks, this was, however, a multi-centre study with participants from various countries across the sub-Saharan region, thus lending to its external validity. Also, the responses of the participants were statistically significant even though the number of participants did not meet the target sample size. Finally, the use of a self-report instrument could introduce response bias; therefore, results would have to be interpreted with this in mind.

## Recommendations

Although general awareness about CVD prevention was high, continuous medical education may be necessary to promote the knowledge regarding prioritisation of care according to a 10-year CVD risk. This, alongside regular audit of CVD guideline utilisation, and a brief behavioural change counselling training programme for PCPs in all the regions, may help improve lifestyle counselling rates. Even though positive physician lifestyle practices are a necessary part of promoting patient counselling rates, promoting a healthy self-awareness for lifestyle change among those with poor lifestyle practices may still increase the quality and rates of patient counselling for CVD prevention.

## Conclusion

In assessing knowledge of CVD prevention among FPs and FM trainees in SSA, we found that most of the participants had accurate responses for the CVD prevention knowledge items. However, the awareness appears to be partial because fewer participants were aware of recommendations on prioritisation of care according to a 10-year CVD risk. The majority of the participants had optimal lifestyle practices and most of the participants always or usually counselled their patients on nutrition but were less likely to screen or counsel patients on smoking, alcohol abuse, weight management, diabetes hypertension and exercise. Region of practice or training and physician personal lifestyle score were predictive of CVD patient counselling practice.

## Appendices

**APPENDIX 1:** Statistical information for Figure 1 – Distribution of accuracy of responses on cardiovascular event prevention.†

Questionnaire item‡	*χ*^2^	*df*	*p*
Q1	132.8	4	0.01
Q2	58.0	1	0.01
Q3	64.7	1	0.01
Q4	17.2	4	0.01
Q5	315.2	3	0.01
Q6	91.7	1	0.01
Q7	78.3	3	0.01
Q8	95.2	4	0.01
Q9	61.8	1	0.01
Q10	59.9	1	0.01
Q11	40.4	1	0.01
Q12	48.7	4	0.01
Q13	323.3	4	0.01
Q14	96.4	4	0.01
Q15	345.8	4	0.01
Q16	21.3	4	0.01

α = 0.05.

Q6 – obesity; Q7 – alcohol intake; Q8 – smoking and CVD risk; Q9–Q12 – hypertension and CVD risk; Q13–Q15 – diabetes mellitus and CVD risk; Q16 – antiplatelet medication use.

df, degree of freedom.

† , Test statistic: χ^2^ goodness-of-fit test.

‡ , Q1 – prioritisation of care; Q2 – fat intake; Q3 – salt intake; Q4 – vegetables intake; Q5 – physical activity.

## Appendix

**APPENDIX 2:** Statistical information for Figure 2 – Distribution of frequency of lifestyle counselling practices.†

Questionnaire item	*χ*^2^	*df*	*p*
Nutrition	69.3	3	< 0.01
Exercise	68.1	3	< 0.01
Weight Mgt.	76.2	3	< 0.01
Smoking	83.8	4	< 0.01
Alcohol abuse	40.8	3	< 0.01
HTN	113.8	4	< 0.01
Diabetes mellitus	103.6	4	< 0.01

*Source*: Adapted from Voltmer E, Frank E, Spahn C. Personal health practices and patient counseling of German physicians in private practice. ISRN Epidemiol [serial online]. 2013 [cited 2015 May 4]; 1–10. Available from: http://www.hindawi.com/journals/isrn.epidemiology/2013/176020/

*α* = 0.05.

HTN, hypertension; Mgt., management; *df*, degree of freedom.

† , Test statistic: *χ*^2^ goodness-of-fit test.

## Appendix

**APPENDIX 3:** Statistical information for Figure 3 – Distribution of personal lifestyle habits of the participants.†

Questionnaire item‡	*χ*^2^	*df*	*p*
L1	86.6	2	0.01
L2	148.9	4	0.01
L3	88.5	4	0.01
L4	100.1	1	0.01
L5	73.8	3	0.01
L6	33.2	3	0.01
L7	101.5	3	0.01
L8	296.5	2	0.01
L9	226.1	5	0.01
L10	155.1	3	0.01
L11	91.9	4	0.01
L12	105.6	4	0.01
L13	49.9	4	0.01
L14	202	2	0.01

*Source*: Adapted from Voltmer E, Frank E, Spahn C. Personal health practices and patient counseling of German physicians in private practice. ISRN Epidemiol [serial online]. 2013 [cited 2015 May 4]; 1–10. Available from: http://www.hindawi.com/journals/isrn.epidemiology/2013/176020/

*α* = 0.05.

*df*, degree of freedom.

† , Test statistic: *χ*^2^ goodness-of-fit test.

‡ , L1 – diet; L2 – exercise frequency; L3 – exercise duration; L4 – salt; L5 – water intake; L6 – coffee; L7 – alcohol; L8 – smoking; L9 – sleep duration; L10 – red meat; L11 – regular check-up; L12 – cholesterol check; L13 – blood glucose check; L14 – have a personal doctor.

## APPENDIX 4: Online survey questionnaire

### Demographics/Baseline data:

1. Kindly state your age (in years): __________
2. Kindly indicate your gender:  Male [ ] Female [ ]
3. What best describes your marital status?

### Married [ ] Single [ ] Separated [ ] Divorced [ ] Widowed [ ]

4. Kindly indicate your nationality (drop-down list)
5. Indicate the country in which you are currently practicing or undergoing your training
6. What kind of population demographic does your practice/training centre serve?

### Urban [ ] Rural [ ] Other (indicate): _____________________________________

7. What is your religion?

### Christianity [ ] Islam [ ] African traditional religion [ ] Decline [ ] Others:__________

8. What is your average monthly income_______________ (Kindly state the currency as well)
9. How many hours do you work in a week?_______________ (Kindly state an absolute value in hours)
10. Which of the following describes your current status:
  - A Family Medicine Resident/Trainee  [ ]
  - A Family Physician (i.e. completed the PG training/Residency training in family medicine)  [ ]

### If you are a Family Medicine Resident/Trainee, kindly respond to Q 11, if not skip to Q 12

11. For how long have you been in the training programme?
  - Indicate number if in years_________________
  - If in months, indicate number here ____________________
12. If you are a Family Physician, how many years has it been post-qualification ________________?
13. Please indicate (i.e. tick against each option), if any of the following are readily available in your consulting room or within 10 m from your consulting room:
  Blood pressure measurement [ ], Weighing scale and tape [ ], Glucometer [ ],
  Blood cholesterol measurement or a point for sample collection [ ],
  Urine glucose measurement (dipstick) [ ], Urine albumin measurement (albustix) [ ]
14. Could you indicate (i.e. tick against an option), some barriers you face in screening for cardiovascular disease in your practice?
  Lack of Time [ ], Ineffective training [ ], No compensation [ ], Language barrier [ ]
  Lack of screening equipments [ ], Facilities available but process of screening is stressful [ ],
  The screening tests are expensive [ ], Other (please indicate…………………………………)
15. From the list of health conditions provided below, kindly tick those you currently suffer from:
  Hypertension [ ], Diabetes [ ], Dyslipidaemia [ ], Obesity [ ]
16. Is any of members of your family suffering from at least one of the diseases listed in Q15 above?
  Yes [ ] No [ ]
17. Have you suffered from any of the following diseases?
  **Table:**
  Cerebrovascular disease (Stroke)	Yes [ ] No [ ]
  Coronary heart disease (Angina, MI, etc.)	Yes [ ] No [ ]
  Pulmonary embolism/Deep venous thrombosis	Yes [ ] No [ ]
  Peripheral arterial disease	Yes [ ] No [ ]
18. Have any of your family members suffered from any of the diseases listed in Q17? Yes [ ] No [ ]

### Physician health-related lifestyle practices

19. How often do you eat salad/raw vegetables/fresh fruits?
  - ≥ once a day
  - ≥ once a week (but less than once every day)
  - ≥ once a month (but less than once every week)
  - Rarely/Never
20. How often do you exercise?
  - >5 days in a week
  - 4–5 days in a week
  - 2–3 days in a week
  - 1 day in a week
  - Not at all
21. What is your total exercise duration in a day (in minutes)?
  - >40 [ ]
  - 30–40 [ ]
  - 20–30 [ ]
  - 10–20 [ ]
  - <10 [ ]
22. Do you add raw salt to your meals? Yes [ ] No [ ]
23. How would you estimate your daily drinking water intake to be?
  - <500 mL [ ]
  - 500–1000 mL [ ]
  - 1000–1500 mL [ ]
  - >1500 mL [ ]
24. How often do you take coffee/black tea?
  - ≥ once a day
  - ≥ once a week (but less than once every day)
  - ≥ once a month (but less than once every week)
  - Rarely/Never
25. How often do you drink alcohol?
  - ≥ once a day
  - ≥ once a week (but less than once every day)
  - ≥ once a month (but less than once every week)
  - Rarely/Never
26. Concerning the use of cigarettes, which best describes you?
  - I currently smoke
  - I used to smoke
  - I have never smoked
27. For how long do you sleep each day (hours)
  - <6 [ ]
  - 6 [ ]
  - 7 [ ]
  - 8 [ ]
  - >8 [ ]
28. In a typical week, how often do you eat red meat?
  - ≥3 times a day
  - Once a day
  - Once in 2–3 days
  - Rarely/Never

### Physician Personal Clinical Preventive Measures

29. How often do you undergo a physical check-up?
  - <1 year
  - 1 to <2 years
  - 2 to <3 years
  - 3 to <4 years
  - 4 to <5 years
  - ≥5 years
  - Never
30. How often do you take your Cholesterol measurement?
  - <1 year
  - 1 to <2 years
  - 2 to <3 years
  - 3 to <4 years
  - 4 to <5 years
  - ≥5 years
  - Never
31. How often do you take a diabetes screening test?
  - <1 year
  - 1 to <2 years
  - 2 to <3 years
  - 3 to <4 years
  - 4 to <5 years
  - ≥5 years
  - Never
32. Do you have a Personal doctor? Yes/No

### Awareness of Primary CVD prevention methods

34. According to WHO/ISH guidelines, when resources are limited, individual counselling and provision of care may have to be prioritised according to a__________ risk of cardiovascular event.
  - 5 years
  - 10 years
  - 15 years
  - 20 years
  - I do not know

### Concerning Dietary Changes

35. Total fat intake should be reduced to about 30% of calories, saturated fat to less than 10% of calories and most dietary fat should be polyunsaturated or monounsaturated (10% – 15% of calories).
  **True [ ]** False [ ]
36. All individuals should be strongly encouraged to reduce daily salt intake by at least one third and if possible, to <5 g per day.
  **True [ ]** False [ ]
37. The recommended daily allowance of fruits and vegetables for all individuals is _____?
  - 200 g
  - 300 g
  - 400 g
  - 500 g
  - 600 g

### Concerning Physical Activity

38. The recommended duration of moderate physical activity that all individuals are encouraged to take per day is ____?
  - At least 30 min
  - At least 40 min
  - At least 50 min
  - At least 1 h

### Concerning Weight Control

39. All individuals who are overweight or obese should be encouraged to lose weight through:
  - A combination of increased-energy diet and increased physical activity
  - A combination of a reduced-energy diet and increased physical activity
  - A reduced-energy diet alone
  - Increased physical activity alone
  - A combination of reduced-energy diet and reduced physical activity

### Concerning Alcohol Intake

40. A male bank executive on follow-up at your clinic for hypertension reports that he consumes alcohol. At what level of his consumption would you advise him to reduce alcohol intake?
  - More than 2 units of alcohol per day
  - More than 3 units of alcohol per day
  - More than 4 units of alcohol per day
  - More than 5 units of alcohol per day

### Concerning Smoking

41. For smokers with a 10-year CVD risk of 20% – 30%, which will you consider the most appropriate, from the treatment options below:
  - Nicotine replacement therapy only
  - Nortriptyline only
  - Amfebutamone and Nortriptyline only
  - Nicotine replacement, Nortriptiline or Amfebutamone
  - I do not know.

### Concerning Antihypertensive Drugs

42. All individuals with blood pressure at or above 160/100 mmHg, or lesser degree of raised blood pressure with target organ damage, should have drug treatment and specific lifestyle advice to lower their blood pressure and risk of cardiovascular disease.
  - Strongly agree
  - Agree
  - Unsure
  - Disagree
  - Strongly disagree
43. All individuals with blood pressure below 160/100 mmHg, or with no target organ damage need to be managed
  - According to the neurovascular risk
  - According to a 10-year cardiovascular risk
  - According to the renovascular risk
  - According to the endocrine risk
  - According to a case-by-case basis

### Concerning Antihypertensive Drugs

44. For a 10-year risk of CVD event ≥30% in individuals with persistent blood pressure ≥130/80 mmHg; a low-dose thiazide-like diuretic, ACE inhibitor or calcium channel blocker is recommended as first-line therapy.   True/False
45. For a 10-year CVD risk of ≥30% in individuals with persistent blood pressure ≥130/80 mmHg, tick the recommended first-line therapy from the options provided below:
  **Table:**
  a. Low-dose thiazide-like diuretic	[ ]
  b. An ACE Inhibitor	[ ]
  c. Calcium channel blocker	[ ]
  d. All of the above	[ ]
  e. None of the above	[ ]

### Concerning Lipid-Lowering Drugs (Statins)

46. Mr Abu is a 67-year old pensioner who is hypertensive and a diabetic. He presented with a result showing a total cholesterol of 12 mmol/L (464 mg/dL). What single best option below will you consider:
  - He should be counselled to follow a lipid-lowering diet only
  - A prescription for statins to lower the risk of cardiovascular disease is preferable
  - Lipid-lowering diet and statins should be included in the treatment option for Mr Abu
  - The results provided above does not need a special diet or statins
  - Focus should be on glycaemic and blood pressure control only
47. Concerning Mr Abu above, the treatment choice should be based on an estimated cardiovascular risk. This is______
  - Always true
  - Sometimes true
  - Not applicable for Africa
  - Never true
  - I do not know

### Concerning Hypoglycaemic Drugs

48. Individuals with persistent fasting blood glucose >6 mmol/L despite diet control should be given
  - Metformin
  - Glibenclamide
  - Pioglitazone
  - Arcabose
  - I do not know

### Concerning Antiplatelet Drugs

49. At what level of cardiovascular risk should low-dose Aspirin be prescribed?
  - <10%
  - 10% – 20%
  - 20% – 30%
  - ³30%
  - I do not know

### CVD Counselling Practice:

50. How often do you counsel your patients on nutrition?
  - Always
  - Usually
  - Sometimes
  - Rarely
  - Never
51. How often do you counsel your patients about regular exercise?
  - Always
  - Usually
  - Sometimes
  - Rarely
  - Never
52. How often do you counsel your patients on weight management?
  - Always
  - Usually
  - Sometimes
  - Rarely
  - Never
53. How often do you provide counselling for patients who need to quit smoking?
  - Always
  - Usually
  - Sometimes
  - Rarely
  - Never
54. How often do you provide counselling for patients who abuse alcohol?
  - Always
  - Usually
  - Sometimes
  - Rarely
  - Never
55. How often do you counsel your patients on screening for high blood pressure?
  - Always
  - Usually
  - Sometimes
  - Rarely
  - Never
56. How often do you counsel your patients on cholesterol screening and management?
  - Always
  - Usually
  - Sometimes
  - Rarely
  - Never
57. How often do you counsel your patients on screening for diabetes?
  - Always
  - Usually
  - Sometimes
  - Rarely
  - Never

### Thank you for your time.

_____________________________________________________________________________________

## Appendix 5: Consent form

### Part I: Information Sheet

Dear Sir/Madam,

I am a family physician with AfriWon which stands for the African Organization for Young and Future General Practitioners (GPs)/Family Physicians (FPs) under the aegis of the African Region of World Organization of Family Doctors (WONCA Africa).

I intend to find out about your knowledge, personal practices and counselling practices as regards lifestyle and cardiovascular disease.

You will be asked to provide information on your demographics, some health-related lifestyle habits, some personal preventive measures, your awareness of primary CVD prevention method and your CVD counselling practice. There are 56 questions in total. We estimate you may need up to 30 min or slightly more to complete the survey. Please try and answer all the questions if you can.

The results of this project will be reported in a reputable journal and may be presented in a WONCA- or WONCA-affiliated conference.

Your participation is entirely of your own free will and you can withdraw from this study at any time you like without explanation. You have the right to refuse to answer any question you do not want to respond to.

Any information collected will remain confidential. Your name will not be attached to any published results. Only I and the coauthors will have access to the data.

Whether you join or refuse to join will have no effect on your employment status/grade level/salary level/rights and privileges in your training institution or academic college.

Thank You.

### Part II: Certificate of Consent

I have read the foregoing information and I have had the opportunity to ask questions about it and any questions that I have asked have been answered to my satisfaction. I consent voluntarily to join as a participant in this study.

Name of participant:

Signature of participant:       Researcher:

Date:           Phone:
